# Autism as a disease of the synapse: search for mechanistic insight

**DOI:** 10.1186/2193-1801-4-S1-L22

**Published:** 2015-06-12

**Authors:** Hannelore Ehrenreich

**Affiliations:** Clinical Neuroscience, Max Planck Institute of Experimental Medicine, Göttingen, Germany

**Keywords:** Autism, Synapse, Ambra1

Autism spectrum disorders (ASD) are heterogeneous, heritable neurodevelopmental conditions, affecting ~0.5% of the population across cultures, with a ~4:1 male/female ratio. ASD are characterized by social interaction and communication deficits, restricted interests, repetitive behaviors, and reduced cognitive flexibility. Causes likely converge at the synapse, as shown by mutations of synaptic genes or mutations causing quantitative alterations in synaptic protein expression. Neuroligin4 (NLGN4X) mutations are among the most frequent causes of heritable ASD. But monogenetic forms altogether account for 1200 schizophrenic subjects and validated it in Asperger autists. We hypothesized that a coincidence of unfortunate normal variation in synaptic or synapse-regulating genes rather than mutations underlies most autistic phenotypes. We identified ‘proautistic’ variants in synaptic genes, which in aggregate are associated with high autism severity. A transcranial magnetic stimulation study on respective individuals revealed enhanced glutamatergic and GABAergic activity. IPS-derived cortical neurons from these subjects are now functionally characterized.

